# The Relationship Between Primary Colorectal Cancer Tumour Characteristics and Patient Outcomes Following Surgery for Oligometastatic Pulmonary Metastases: A Single-Centre Retrospective Study

**DOI:** 10.3390/cancers18193080

**Published:** 2026-09-22

**Authors:** Anam Asad, Marcus Taylor, Thomas Craze, David Woolf, Gokul Raj Krishna, Sayan Biswas, Asmita Singhania, Matt Evison, Kandadai Rammohan, Omer Aziz, Meera Patel

**Affiliations:** 1 Colorectal & Peritoneal Oncology Centre, The Christie NHS Foundation Trust, Manchester M20 4BX, UK; 2 Department of Cardiothoracic Surgery, Manchester University Hospital NHS Foundation Trust, Wythenshawe Hospital, Manchester M23 9LT, UK; 3 Department of Clinical Oncology, The Christie NHS Foundation Trust, Manchester M20 4BX, UK; 4 Department of Thoracic Oncology, Manchester University Hospital NHS Foundation Trust, Wythenshawe Hospital, Manchester M23 9LT, UK

**Keywords:** colorectal cancer, metastasis, pulmonary metastases, pulmonary metastasectomy

## Abstract

**Background**: Colorectal cancer (CRC) is the third most common cancer globally. Approximately 20% present with metastatic disease, most commonly to the liver and lung. Pulmonary metastasectomy (PM) is the most considered therapeutic option for oligometastatic colorectal lung metastases, though evidence is predominantly retrospective. This study aims to understand the relationship between primary colorectal cancer tumour characteristics and survival in patients undergoing PM. **Methods**: This single-centre retrospective study included 372 consecutive patients undergoing PM at Manchester University Foundation Trust (2011–2021). Data was extracted from prospectively maintained clinical databases. Overall survival was calculated from the date of PM. Kaplan–Meier log-rank and Cox proportional hazards regression analyses identified prognostic factors. **Results**: Two-thirds of the patients included were over the age of 65 (65%), and 63% were male. The median follow-up was 48 months, with a median survival of 69 months. On univariate analysis, age ≥ 65, advanced TNM, T stage, right-sided primary tumour location, lymphovascular invasion, and KRAS mutation were associated with reduced overall survival. Upon multivariate analysis, age ≥ 65 (HR 2.60, 95% CI 1.14–5.91, *p* = 0.023) and lymph node yield ≥ 12 (HR 0.38, 95% CI 0.17–0.83, *p* = 0.016) were independently associated with survival. **Conclusions**: Primary tumour characteristics are important determinants of outcomes after PM. Older age, right-sided primary tumour location, inadequate lymph node yield and KRAS mutation are associated with survival, highlighting the need for refined patient selection. These findings support integrating clinical, pathological, and molecular factors to aid decision-making in patients with colorectal pulmonary metastases.

## Simple Summary

Pulmonary metastasectomy (PM) is commonly used to treat selected patients with colorectal cancer that has spread to the lungs; it remains unclear how primary colorectal tumour features influence the outcome after PM. This study examined whether characteristics of the original colorectal tumour were associated with survival in 372 patients who underwent PM. Older age, more advanced primary tumour stage, right-sided tumour location, lymphovascular invasion, and KRAS mutation were associated with poorer overall survival, while an adequate lymph node yield at primary colorectal surgery was associated with improved survival. Older age and lymph node yield remained independently associated with survival after accounting for other tumour characteristics. These findings demonstrate that characteristics of the patient and original colorectal tumour provide useful prognostic information and could contribute to more individualised multidisciplinary decision-making when considering treatment options for colorectal lung metastases.

## 1. Introduction

Colorectal cancer (CRC) is the third most common cancer worldwide. In the United Kingdom, there are over 48,000 new cases each year [1]. Approximately 20% of new CRC diagnoses will have metastatic disease at diagnosis. The management of oligometastatic disease over the last decades has evolved, from a time when metastatic disease would dictate a palliative pathway to current practice where oligometastatic disease can be managed with curative intent. The most common site of metastasis is the liver, followed by the lung [2]. Incidence of synchronous metastases is variable, with liver metastasis reported at 14–19% [3] and lung metastasis at 11% [4].

Current consensus guidelines from the European Society of Medical Oncology and the National Comprehensive Cancer Network Guidelines in the United States for management of pulmonary metastasis have remained relatively unchanged since the 1950s [5,6]. Pulmonary metastasectomy (PM) for colorectal pulmonary metastases is the most considered therapeutic option, with potential for a curative outcome [6,7]. Indications for surgical resection of pulmonary metastases in CRC include technical feasibility with negative margins, typically oligometastatic disease with ≤3–5 metastases, adequate pulmonary reserve, patient fitness, and absence of distant metastases (with the exception of resectable liver metastases) [7,8]. However, there remains uncertainty surrounding the potential survival benefit gained from PM. Much of the evidence reporting survival benefit from surgery is retrospective in nature and therefore lacking a comparable control population. For example, a large meta-analysis including 25 non-randomised studies with 2925 patients reported an overall survival benefit of 41% in patients undergoing PM [9]. A systematic review and meta-analysis including 115 studies and 13,294 patients looked at the prognostic significance of preoperative factors, reporting overall 5-year survival after surgical resection of PM at 54% [10].

The multi-centre PulMiCC trial (Pulmonary Metastasectomy versus Continued Active Monitoring in Colorectal Cancer) was the first to investigate the evidence base for PM, reporting a 9% increase in 5-year overall survival in those undergoing PM compared to those undergoing active surveillance, a considerably smaller survival benefit than that reported in previous studies [9,11]. Despite being limited by poor recruitment and early trial closure, PulMiCC challenges the long-held assumption that PM confers a significant survival benefit compared with no local treatment. Importantly, the ‘no surgery’ group had better survival than historically assumed, at 29% 5-year overall survival.

Several factors have been associated with a better outcome following PM. These include normal CEA pre- and post-metastasectomy [12], solitary metastasis [10,12,13], node-negative thoracic disease [10,13], absence of extra-thoracic disease, size < 2 cm, primary T stage < T4, and absence of p53 mutation [10]. The presence of KRAS mutation has been independently associated with poor survival following PM; however, a full understanding of how molecular markers provide prognostic information in the context of PM is limited [14,15]. Whilst several factors have been shown to be associated with outcome following PM, it is important to understand that these do not predict benefit from PM versus surveillance or treatments such as SABR.

There has been significant evolution in the treatment of oligometastatic colorectal cancer over the last few decades, with advances in systemic treatments, local ablative therapies, surgery, and imaging modalities. PM is offered to a heterogeneous group of patients, and equally, patients with seemingly similar primary tumours can experience markedly different patterns of metastatic spread and clinical trajectories. Therefore, a better understanding of how patient- and tumour-specific characteristics influence outcomes after PM may aid clinical decision-making. Here, we aim to investigate how features of the original colorectal primary tumour can refine the selection of patients undergoing PM.

## 2. Methods

In this single-centre retrospective study, all consecutive patients undergoing any form of lung resection (including sublobar wedge resection, segmentectomy, lobectomy, and pneumonectomy) at Manchester University Foundation Trust between November 2011 and May 2021 for PM were included. Patient and surgical data were collected retrospectively from a prospectively maintained Dendrite clinical platform, where data are entered at the time of surgery. Further data related to the primary tumour were retrieved from The Christie NHS Foundation Trust Clinical Web Portal system. Age was dichotomized into two groups (<65 and ≥65 years) according to the World Health Organization definition of elderly, and given that most colorectal cancer cases are diagnosed in patients aged 65 or older. Lymph node yield, defined as the number of lymph nodes resected during primary colorectal tumour resection, was categorised as a binary variable using a threshold of 12 lymph nodes, in accordance with American Joint Committee on Cancer guidelines that recommend a minimum yield of 12 for accurate staging and oncologic clearance.

Patients with colorectal pulmonary metastases remained under the care of the colorectal surgical and oncological teams, and hence decision-making to refer for surgical consideration of PM was primarily driven by colorectal multidisciplinary team (MDT) meetings. In general, patients were referred only if complete surgical clearance at the time of index colorectal surgery was achieved and had no more than three pulmonary metastases. However, if patients were younger or otherwise deemed to be suitable for a more aggressive approach, those with a short disease-free interval, the presence of limited extra-thoracic metastatic disease, and more than three pulmonary metastases were considered. After patients were referred by the colorectal MDT, they were seen by a consultant thoracic surgeon to assess their fitness for surgery (i.e., Performance Status score 0–1, with no other significant comorbidities and acceptable pulmonary function tests) and then subsequently discussed in a quorate lung cancer MDT to ensure the wider lung cancer team felt that PM was appropriate in each case. All decisions regarding systemic anti-cancer therapy, either before or after PM, were also made by the colorectal MDT across a wide geographical area, and hence this information was not available.

The general approach of the unit was to take a sublobar approach wherever anatomically feasible in order to preserve lung parenchyma, given that previous evidence suggests that anatomical resection conveys no oncological superiority. However, in cases where metastases were large, where several metastases were present within the same lobe, or when the anatomical location of a metastasis would complicate or prevent non-anatomical resection from being performed (i.e., if a lesion was deep within the lobe or immediately adjacent to a vascular structure), anatomical lobectomy was performed to facilitate complete resection. Over time, as the unit’s segmentectomy experience grew, some surgeons opted to perform segmentectomy for appropriately located lesions. The aim was to achieve a complete resection with adequate margins whilst maintaining as much healthy lung tissue as possible. There was a trend towards more aggressive and extensive resections for younger patients with greater physiological reserve in comparison to patients who were older with a greater number of comorbidities or frailty.

All data were stored in the Northwest Clinical Outcomes Research Registry (NCORR) (IRAS 340354). The NCORR database has full ethical approval from the regional Ethics Committee of the Health Research Authority. This project was approved by the NCORR steering committee, and individual patient consent was waived due to anonymisation of the data prior to use.

Statistical analysis was performed using SPSS version 29 (IBM SPSS, Inc., Chicago, IL, USA). Survival (months) was calculated from the date of surgery for PM until death or the time of the most recent follow-up. Death records were complete until 1 May 2023; this acted as the censor date for survival analysis. Overall survival was examined using Kaplan–Meier log-rank survival analysis and univariate Cox proportional hazards regression to calculate hazard ratios (HR) and 95% confidence intervals. Variables found to be statistically significant (*p* < 0.05) upon univariate analysis were entered into a Cox regression multivariate model using a backward conditional method. For patients who had undergone subsequent surgical resection (i.e., more than one PM), only the first PM was included in the survival analysis. Repeat entries (*n* = 119) were excluded due to potential impact on survival analysis.

## 3. Results

### 3.1. Patient Characteristics

In total, 372 patients who underwent PM between November 2011 and May 2021 were included in the final analysis (Figure 1). Patient demographic, clinical, and pathological features are demonstrated in Table 1. The median time from index colorectal surgery to PM was 22 months (interquartile range 13–35 months). In those with rectal cancer, 123 (69%) underwent neoadjuvant chemoradiotherapy and 119 (74%) underwent adjuvant chemotherapy. In those with colonic tumours, 11 (9%) received neoadjuvant chemotherapy and 101 (81%) received adjuvant chemotherapy.

Two-thirds of the patients included were over the age of 65 (65%), and 63% were male. In total, 189 (51%) had a primary rectal tumour, 83 (22%) had left-sided tumours, and 56 (15%) had right-sided tumours. In terms of disease stage at presentation, 29 (8%) were TNM stage 1, 97 (27%) stage 2, 183 (52%) stage 3, and 46 (12%) stage 4.

The median disease-free interval was 22.4 months (IQR 12.8–34.9). The median follow-up of all patients was 48 months (IQR 34–77 months), calculated as the median time from PM to death or the last follow-up (1 May 2023). The median survival was 69 months, with 181 deaths overall.

### 3.2. Metastasectomy Characteristics

In total, 226 (61%) of patients had a sublobar wedge resection, 21 (5%) anatomical segmentectomy, 124 (33%) lobectomy, and one patient underwent pneumonectomy. Surgery was performed via thoracotomy in 235 (63%) cases, and via video-assisted thoracoscopic surgery (VATS) in 137 (37%) patients. In terms of location, 190 (51%) had right-sided surgery and 12 (3%) underwent synchronous bilateral resection. Mediastinal lymph node harvesting was performed in 87 (23%) cases. The number of metastases resected ranged from one to nine, with a modal value of 1. Data regarding CEA levels, completeness of pulmonary resection, thoracic nodal status, and perioperative systemic anti-cancer treatment were not available.

### 3.3. Survival Analysis

Upon univariate analysis, age (HR 1.47, 95% CI 1.06–2.03, *p* = 0.018), TNM stage (HR 1.30 (1.07–1.58, *p* = 0.002), T stage (HR 1.24, 95% CI 1.00–1.55, *p* = 0.007), lymphovascular invasion (HR 1.79, CI 1.05–3.04, *p* = 0.029), and presence of KRAS mutation (HR 1.61, CI 1.00–2.60, *p* = 0.047) were associated with a reduction in overall survival (Figure 2). Primary tumour site was associated with overall survival (*p* = 0.038). Compared with right-sided colon cancer, left-sided primary tumours were associated with improved survival (HR 0.56, 95% CI 0.35–0.89, *p* = 0.015), as were rectal primary tumours (HR 0.66, 95% CI 0.44–0.99, *p* = 0.043). Lymph node yield ≥ 12 (HR 0.63, 95% CI 0.40–0.97, *p* = 0.034) was associated with improved survival. Sex, neoadjuvant treatment, adjuvant treatment, resection of liver metastases, nodal stage, tumour differentiation, and BRAF, NRAS, and PIK3CA mutational statuses were not associated with overall survival on univariate analysis. All univariate-significant variables (age, TNM, T stage, primary tumour location, lymph node yield, lymphovascular invasion, and KRAS mutational status) were included in a multivariate analysis. Upon multivariate analysis, both age (HR 2.91, 95% CI 1.22–6.89, *p* = 0.023) and lymph node yield (HR 0.38, 95% CI 0.17–0.83, *p* = 0.016) were independently associated with overall survival (Table 2).

## 4. Discussion

This study of 372 patients undergoing PM for oligometastatic colorectal lung metastases demonstrates that key primary tumour features are prognostic in terms of overall survival. This highlights the need to consider primary tumour and host characteristics when refining patient selection for PM. The present study identified older patients (>65 years) and a lymph node yield ≥ 12 as independent predictors of survival after PM. Upon univariate analysis, right-sided primary tumour location, TNM stage, T stage, lymphovascular invasion, and KRAS mutation were also associated with reduced overall survival. Patients with right-sided primary tumours had worse overall survival outcomes when compared to those with left-sided or rectal cancer. These findings underscore the importance of patient- and tumour-specific characteristics in risk-stratifying patients for PM. In the present study, although liver resection was not associated with outcome, previous studies suggest that the presence of extra-thoracic and liver metastases is strongly associated with survival in patients undergoing PM [10].

The present study identified lymph node yield ≥ 12 as an independent predictor of improved overall survival after PM. Lymph node yield is widely regarded as a benchmark for the quality of colorectal cancer surgery: inadequate surgical resection or pathological examination may lead to understaging and therefore undertreatment, with subsequent prognostic implications. In the present study, the presence of primary tumour lymphovascular invasion was associated with reduced overall survival upon univariate analysis, although it was not retained as an independent predictor upon multivariate analysis. This is consistent with other studies evaluating the prognostic role of lymphovascular invasion after primary tumour surgery [16,17]. Lymphovascular invasion may reflect the biological propensity of primary tumours to spread to regional and distant nodes. The results of the present study and other published evidence suggest it to be an important prognostic factor which should be integrated into prognostic models to guide decision-making [17]. Primary tumour location has been investigated in several studies examining outcomes following PM. Rectal primary tumour location has been associated with worse survival outcomes [18,19]. The present study suggests right-sided primary tumour location is associated with worse overall survival after PM, a finding that is consistent with general survival outcomes in patients undergoing systemic anti-cancer treatment for metastatic colorectal cancer.

Whilst surgery has traditionally been the most considered treatment option, the PulMiCC trial, despite its limitations, challenges the dogma that PM provides a significant survival benefit. It also suggests that some patients with indolent disease may not require immediate surgery. Indeed, there appears to be a need for better patient selection for PM [11]. Patients selected for PM are a highly selected group; as such, any analysis, including that of the present study, should be aware that PM itself may confer a survival advantage.

Simultaneously, the emergence of stereotactic ablative therapy offers an alternative treatment modality for oligometastatic disease. The 2019 SABR-COMET trial, which included multiple tumour types, demonstrated SABR improved overall survival by 22 months compared to standard palliative therapy in patients with oligometastatic disease [20]. Furthermore, a recent large systematic review (including 29,932 patients who underwent metastasectomy and 1381 who received SABR) demonstrated a comparative OS rate of 52.2% vs. 45% but an improvement in local control with surgery [21]. These data would support the utilisation of SABR in older patients who may have a worse outcome, as suggested by the results of the present study, and hence the funding of randomised studies should be encouraged.

Colorectal cancer is indeed a heterogeneous disease. The use of molecular profiling in metastatic colorectal cancer is routine across the UK. Although molecular features do not routinely inform surgical decision-making, the present study suggests patients with a KRAS mutation have worse outcomes. In a retrospective study by Ghidini et al., evaluating the prognostic role of KRAS, BRAF and PIK3CA mutations and loss of PTEN in surgically resected metastases, KRAS mutation was associated with poorer survival compared to the wild-type group on both univariate and multivariate analyses [15]. KRAS mutation has also been associated with early pulmonary recurrence and a more diffuse pattern of metastasis [22]. Therefore, there may be a role for utilising KRAS status to inform decision-making around surgery and whether less invasive options such as SABR may be more appropriate.

The present study is a large, single-centre cohort, with long-term follow-up providing robust survival data. Although data collection is comprehensive from a prospectively maintained database, the study is limited by its retrospective design. Selection bias arises from including patients undergoing PM who may represent a cohort with fewer comorbidities and favourable tumour biology. The present study had gaps in molecular data. Markers such as BRAF, NRAS, PIK3CA, and MSI/MMR were not consistently available, which therefore limits our ability to fully evaluate the prognostic role of genomic profiling in this cohort. This can be attributed to a time when comprehensive genomic analyses and MMR/MSI testing were not routinely performed in clinical care. In the era of precision oncology, the present study suggests a need for the development of predictive models that integrate clinical, pathological, and molecular factors for prognostic enrichment in MDT decision-making. Additional tools, such as liquid biopsies and circulating tumour DNA, may have a role in decision-making and predicting outcomes for patients with oligometastatic pulmonary metastases.

## 5. Conclusions

In summary, PM remains a cornerstone in the management of oligometastatic colorectal cancer. In the era of precision oncology, optimal patient selection—guided by the integration of host factors, tumour pathology, and molecular markers—is essential for multidisciplinary decision-making.

## Figures and Tables

**Figure 1:**
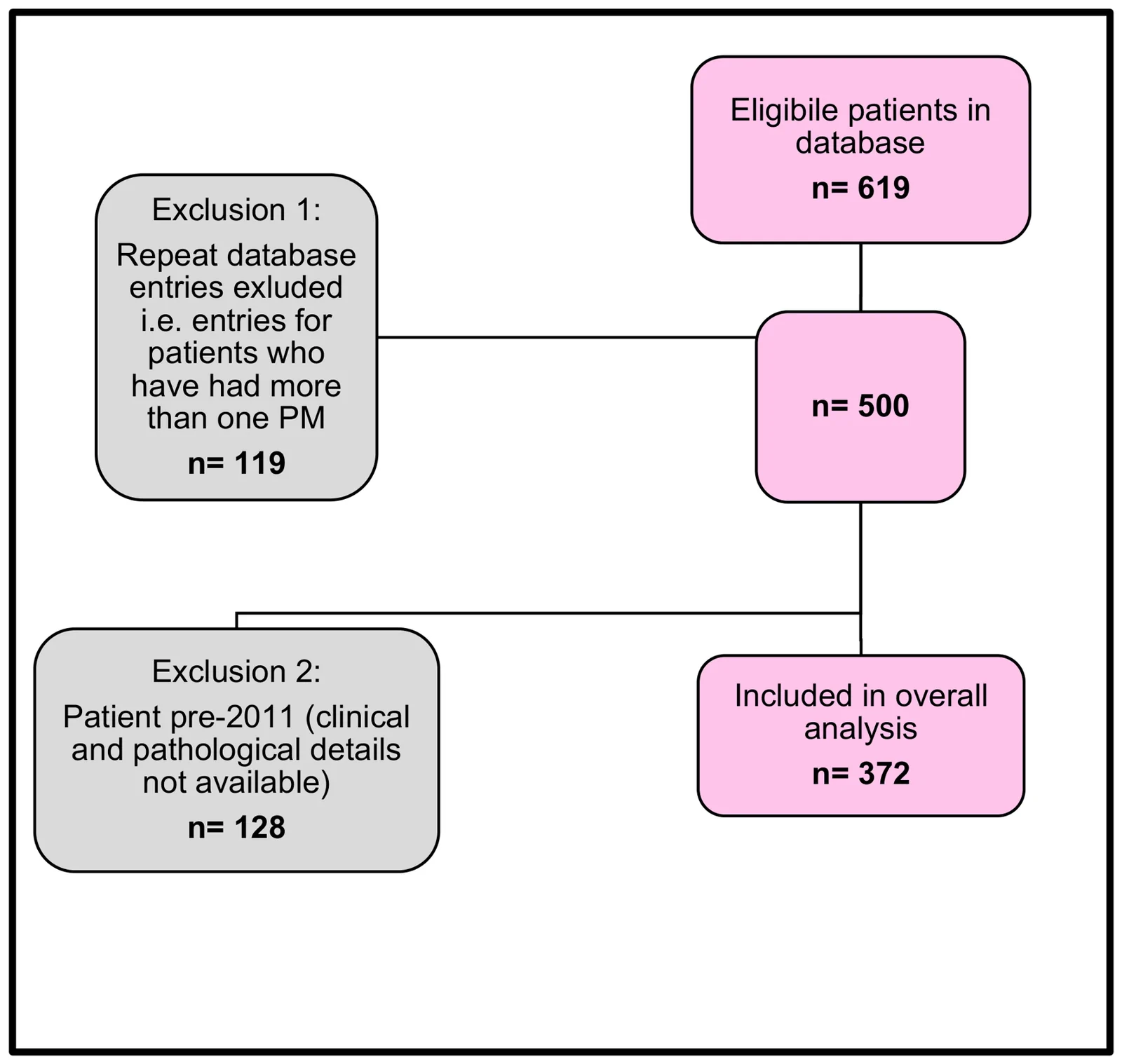
Flow chart detailing patients included in statistical analysis, those who underwent iterative PM for colorectal metastases.

**Figure 2:**
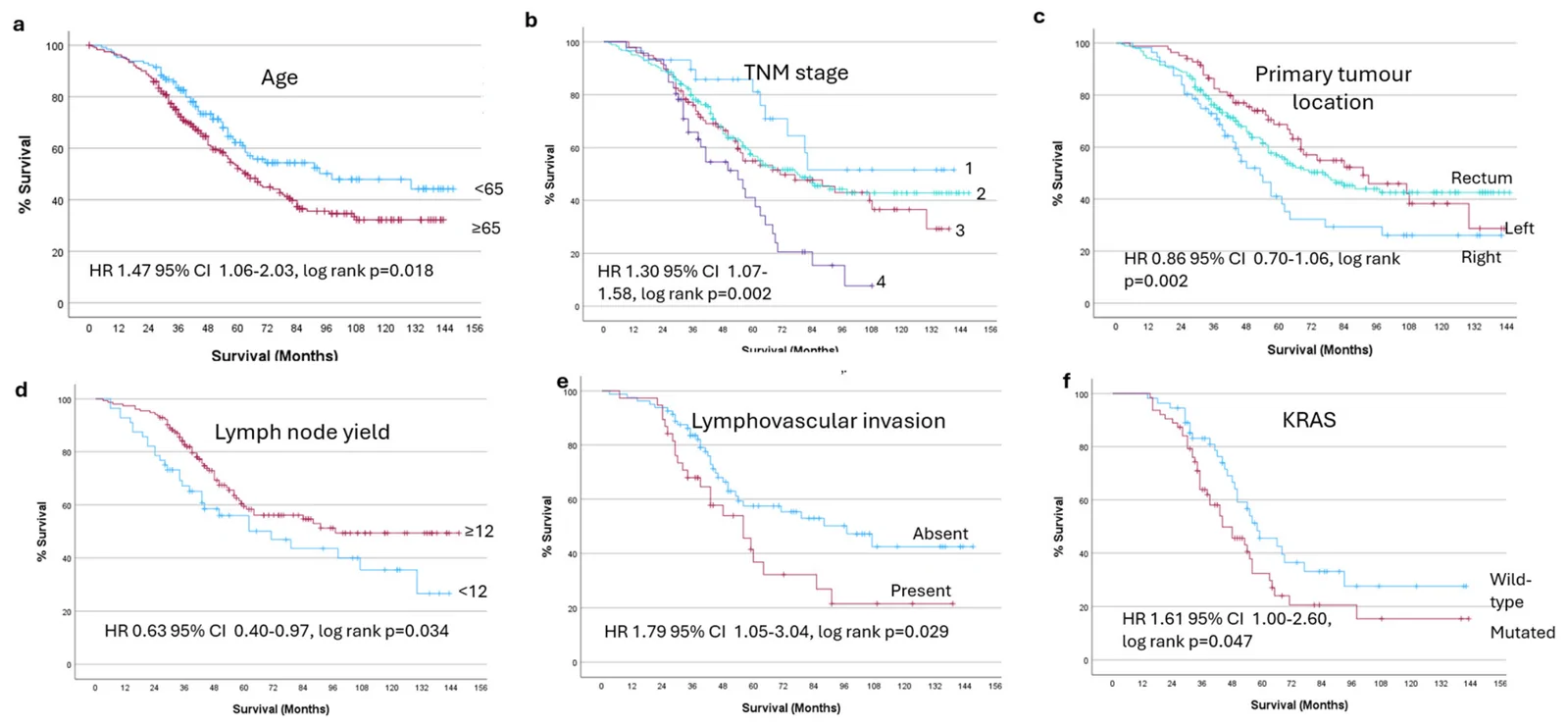
Primary colorectal tumour characteristics and survival in patients undergoing pulmonary metastasectomy. Age > 65 (**a**), TNM stage at diagnosis (**b**), primary tumour location (**c**), lymph node yield (**d**), lymphovascular invasion (**e**), and KRAS status (**f**) were associated with significantly worse overall survival.

**Table 1:** Host and primary tumour characteristics in patients undergoing PM for colorectal cancer.

Host Characteristics	n (%)
Gende r (*n* = 372)	
Female	139 (37)
Male	233 (63)
Age (*n* = 372)	
<65	130 (35)
≥65	242 (65)
ASA (*n* = 372)	
1	22 (6)
2	95 (25)
3	254 (68)
4	1 (1)
Survival Status (*n* = 372)	
Alive	191 (51)
Deceased	181 (48)
Oncological treatment	
Rectal primary	
Neoadjuvant chemoradiation (*n* = 179)	
Yes	123 (69)
No	56 (31)
Adjuvant chemotherapy (*n* = 161)	
Yes	119 (74)
No	42 (26)
Colonic primary	
Neoadjuvant chemotherapy (*n* = 116)	
Yes	11 (9)
No	105 (91)
Adjuvant chemotherapy (*n* = 124)	
Yes	101 (81)
No	23 (19)
Surgical resection for liver metastasis (*n* = 277)	
Yes	93 (34)
No	184 (66)
Cytoreductive Surgery and HIPEC (*n* = 264)	
Yes	7 (3)
No	257 (97)
Tumour Characteristics	
Primary tumour site (*n* = 339)	
Right	56 (17)
Left	83 (24)
Rectum	189 (56)
Synchronous	11 (3)
TNM stage (*n* = 355)	
1	29 (8)
2	97 (27)
3	183 (52)
4	46 (13)
Tumour differentiation (*n* = 217)	
Well-moderate	195 (90)
Poor	22 (10)
Lymph node yield (*n* = 211)	
<12	56 (27)
≥12	155 (73)
Lymphovascular invasion (*n* = 119)	
Present	38 (32)
Absent	81 (68)
Molecular features	
KRAS (*n* = 118)	
Mutated	63 (53)
Wild-type	55 (47)
NRAS (*n* = 93)	
Mutated	3 (3)
Wild-type	90 (97)
BRAF (*n* = 81)	
V600E mutation	9 (11)
Wild-type	72 (89)
PIK3CA (*n* = 71)	
Mutated	8 (11)
Wild-type	63 (89)
Microsatellite status (*n* = 45)	
Microsatellite instability	3 (7)
Microsatellite stable	42 (93)
Metastasectomy characteristics	
Surgical approach (*n* = 372)	
Thoracotomy	235 (63)
Video-Assisted Thoracoscopic Surgery (VATS)	137 (37)
Type of resection (*n* = 372)	
Wedge resection	226 (61)
Segmentectomy	21 (5)
Lobectomy	124 (33)
Pneumonectomy	1 (1)
Side of resection (*n* = 372)	
Left	170 (46)
Right	190 (51)
Bilateral	12 (3)
Mediastinal lymph node sampling (*n* = 372)	
Yes	87 (23%)
No	285 (77%)

**Table 2:** Univariate and multivariate analysis including host and primary tumour features.

Clinicopathological Characteristics	Univariate HR (95% CI)	*p*	Multivariate HR (95% CI)	*p*
Age (<65/≥65)	1.47 (1.06–2.03)	0.018	2.60 (1.14–5.91)	0.023
Sex (Female/Male)	1.33 (0.97–1.81)	0.070		
Neoadjuvant chemoradiotherapy (rectal)	0.96 (0.61–1.52)	0.852		
Adjuvant chemotherapy (colon)	0.88 (0.60–1.30)	0.529		
Surgical resection for liver metastasis	1.02 (0.82–1.27)	0.989		
Primary tumour site				
Right *				
Left	0.56 (0.35–0.89)	0.038		
Rectum	0.66 (0.44–0.99)			
TNM stage	1.30 (1.07–1.58)	0.002		
T stage	1.24 (1.00–1.55)	0.007		
N stage	1.20 (0.96–1.49)	0.209		
Lymph node yield (<12/≥12 *)	0.63 (0.40–0.97)	0.034	0.38 (0.17–0.83)	0.016
Tumour differentiation (well-moderate/poor)	0.95 (0.49–1.81)	0.864		
Lymphovascular invasion (absent/present)	1.79 (1.05–3.04)	0.029		
KRAS (mutant/wild-type)	1.61 (1.00–2.60)	0.047		
BRAF (V600E mutant/wild-type)	0.81 (0.25–2.64)	0.727		
NRAS (mutant/wild-type)	0.81 (0.20–3.34)	0.766		
PIK3CA (mutant/wild-type)	0.94 (0.28–3.08)	0.912		

* Reference group.

## Data Availability

Data available on request due. ethicarestrictions. ical reasons) The data presented in this study are available on request from the corresponding author due to (specify the reason for the restriction).
